# Inspiratory muscle training and aerobic exercise for respiratory muscle strength in myasthenia gravis post-hospitalization- a randomized controlled trial

**DOI:** 10.1186/s12890-025-03733-7

**Published:** 2025-05-27

**Authors:** Chia-Ling Chang, Tien-Pei Fang, Hsin-Mao Tsai, Hui-Chin Chen, Shih-Feng Liu, Hui-Ling Lin, Jui-Fang Liu

**Affiliations:** 1 Department of Respiratory Therapy, Kaohsiung Municipal Fong Shan Hospital - Under the management of Chang Gung Medical Foundation, Kaohsiung, 833 Taiwan; 2 Department of Respiratory Therapy, Kaohsiung Chang Gung Memorial Hospital, Kaohsiung, 833 Taiwan; 3 Department of Respiratory Care, Chang Gung University of Science and Technology, Chiayi, 613 Taiwan; 4 Department of Respiratory Therapy, Chiayi Chang Gung Memorial Hospital, Chiayi, 613 Taiwan; 5 Department of Pharmacy, Chia Nan University of Pharmacy & Science, Tainan, 717 Taiwan; 6 Division of Pulmonary and Critical Care Medicine, Department of Internal Medicine, Kaohsiung Chang Gung Memorial Hospital, Chang Gung University College of Medicine, Kaohsiung, Taiwan; 7 Department of Respiratory Therapy, Chang Gung University, Taoyuan, 333 Taiwan; 8 Chronic Diseases and Health Promotion Research Center, Chang Gung University of Science and Technology, Chiayi, Taiwan

**Keywords:** Myasthenia gravis, Breathing exercises, Muscle strength, Pulmonary function tests, Maximal respiratory pressures, 6-minute walk test

## Abstract

**Background:**

Previous studies have demonstrated the positive effects of long-term inspiratory muscle training (IMT) on inspiratory muscle strength and pulmonary function. However, the benefits of IMT with aerobic exercise (IMT + AE) in patients with myasthenia gravis (MG) remain unclear. This randomized controlled trial aimed to assess the impact of the early, 6-week, moderate-intensity interval IMT + AE on pulmonary function, functional capacity, and respiratory muscle strength in patients with MG post-hospitalization.

**Methods:**

Patients with Discharged MG were randomly assigned to either a control group receiving standard medical management or an intervention group undergoing six-week IMT + AE program. Respiratory status was evaluated using the maximum inspiratory/expiratory pressure (MIP/MEP) and pulmonary function tests. Modified Borg dyspnea scores and a six-minute walk test for functional capacity were also employed.

**Results:**

Fifty-four participants were assigned to either the control (six*n* = 28) or IMT + AE groups (*n* = 26). At 6 weeks, the IMT + AE group showed significant improvements across all parameters, while the control group only showed notable differences in the modified Borg scale scores and walking distance. MIP improvements were 33.8 ± 36.1 cmH_2_O in IMT and 22.1 ± 25.8 cmH_2_O in control groups (*P* = 0.18). The IMT + AE group improvements were more substantial in MEP, modified Borg scale, and 6-minute walk distance, in addition to forced vital capacity (FVC) and FVC % of prediction (0.21 ± 0.24 L and 6.17 ± 6.01%, respectively), while the control group showed decreased volumes (-0.06 ± 0.30 L and − 1.79 ± 9.69%, respectively). FVC improvement was significant with IMT + AE (0.21 ± 0.24 L) vs. reduction in the control group (-0.06 ± 0.3 L; *P* = 0.001).

**Conclusions:**

Implementing six-week moderate-intensity interval IMT + AE effectively enhanced respiratory muscle strength, alleviated dyspnea, improved physical capacity, and increased FVC in patients with MG following hospitalization after discharge.

**Clinical trial registration:**

The study was registered in The Clinical Trials Clinical Trial (NCT06624345||https://www.clinicaltrials.gov/) on October 12, 2024 (retrospectively registered).

**Supplementary Information:**

The online version contains supplementary material available at 10.1186/s12890-025-03733-7.

## Background

Myasthenia gravis (MG), a chronic autoimmune disorder affecting the neuromuscular system, is characterized by rapid fatigue and weakness in voluntary muscles [1]. This condition occurs when the immune system erroneously targets the neuromuscular junction, attacking acetylcholine receptors. The typical manifestations include ptosis, diplopia, dysphagia, dysarthria, and limb weakness. Symptom intensity can fluctuate, is often exacerbated by physical exertion, and can be ameliorated with rest. Diagnostic procedures include clinical evaluation, serological tests for antibodies, and electromyography [2]. Treatments include pharmacological agents to enhance neuromuscular transmission, immunosuppressive therapies, and sometimes surgical interventions, such as thymectomy [3].

Respiratory impairments, including respiratory muscle weakness, dyspnea, ineffective cough, sleep-disordered breathing, aspiration pneumonia, and respiratory failure, are a significant concern in MG. Patients may experience reduced vital capacity, increased susceptibility to respiratory infections, orthopnea, and paradoxical breathing [4]. Enhancing respiratory muscle strength may reduce the risk of respiratory failure in MG patients [5]. Physical exercise training has been shown to improve functional muscle status. When appropriately designed, exercise programs can enhance muscle strength [6]. A 24-week program combining aerobic and resistance strength training yields significant benefits, including improvements in muscular strength, physical function, lung function, walking speed, and symptom severity [7].

Respiratory muscle training has been proven effective for improving respiratory function [8, 9]. Strengthening inspiratory muscles improves cough function, reduces mucus retention, and enhances exercise capacity [10]. Most rehabilitation programs for patients with MG have been designed with 10-week or longer. Studies have shown the long-term benefits of respiratory muscle training for patients with mild-to-moderate MG, particularly those with hypercapnic respiratory failure [6, 8]. However, the efficacy of short-term approaches remains uncertain.

This combination of interval inspiratory muscle training (IMT) and aerobic exercise has been shown to reduce fatigue risk while enhancing respiratory efficiency and muscle endurance [11–13]. The customizable intensity allows for individual adaptation, potentially reducing respiratory complications, and addressing respiratory and overall physical function. This study aimed to evaluate the efficacy of a six-week early intervention program combining moderate-intensity interval IMT and aerobic exercise (IMT + AE) in improving respiratory function, functional capacity, and dyspnea levels in MG patients.

## Methods

### Study population

Approved by the Institutional Review Board of Chang Gung Memorial Foundation (Approval number: 201701697A3), this randomized clinical trial was conducted at Kaohsiung Chang Gung Memorial Hospital, Taiwan, from January 2018 to December 2018. This trial was retrospectively registered in The Clinical Trials Registry (NCT06624345). This study adhered to the CONSORT guidelines and the Declaration of Helsinki. With informed consent from all participants, patients with MG were recruited at their initial outpatient visit following discharge due to acute exacerbation.

All enrolled patients were first diagnosed with MG based on clinical features and serial examinations, including serological positivity for acetylcholine receptor antibodies, electromyography, and response to cholinesterase inhibitors. The inclusion criteria were as follows: (A) willingness to participate, (B) age over 18 years, and (C) ability to complete home-based respiratory muscle and exercise training for six weeks. The exclusion criteria were as follows: (A) presence of Myasthenia Gravis Foundation of America (MGFA) classification I or V; (B) diagnosis of heart, kidney, liver, metabolic diseases, or malignant tumors; (C) presence of unstable hemodynamics; (D) diagnosis of New York Heart Association Class III/IV; and (E) presence of pulmonary disease (FEV_1_/FVC ratio < 0.7, FEV_1_ < 50% predicted), and inability to follow exercise instructions. Patients with MGFA classification I were excluded due to the unlikely involvement of respiratory muscles in disease progression, and those with classification IV were excluded due to the inability to hold the mouthpiece of the IMT device.

### Study protocol

Participants were randomly assigned to the IMT or disease control groups using a computer-generated schedule at discharge from the hospital. The assigned codes were sealed in envelopes and randomly selected by a blinded non-researcher for group allocation with an allocation ratio of 1:1. The study aimed for a sample size of 60 patients in order to achieve a significance level (α) of 0.05 and a power of 80% utilizing G Power statistical software, based on the study conducted by Fregonezi et al., using the mean and standard deviation values of maximal inspiratory pressure (MIP) [13].The control group received standard care without additional interventions, while the training group participated in a comprehensive six-week program including inspiratory muscle strength training and aerobic exercise initiation at enrollment. The control group received standard care consisting of acetylcholinesterase inhibitors (pyridostigmine), immunosuppressive therapy (corticosteroids and azathioprine), and additional supportive medications for non-neurological symptoms with bi-weekly clinical follow-up.

Due to the nature of the training procedure, blinding was only applied to the principal investigator during statistical analysis. Patients in the IMT group underwent intervention using a threshold loading device (Dofin Breathing Trainer, Galmed Corp., I-Lan, Taiwan), set within a pressure range of 5–39 cmH₂O. Participants were instructed to place the device in their mouths, inhale deeply against the applied resistance, hold their breath momentarily, and then remove the device for exhalation. Proper use of the device was reinforced through debriefing after the initial session. Patients were guided to perform the exercise in six sets of five breaths, a total of 30 breaths, twice daily.

The interval training protocol began with a warm-up of five breaths, each incorporating a 3-second breath hold followed by a 10-second break between breaths, with a 2-minute rest period. The endurance training protocol involved five breaths with a 3-second breath hold and a 3-second rest between breaths, repeated across four cycles. A cool-down phase was conducted with a final set of five breaths, allowing a 10-second rest between each. The entire training session lasted for approximately 10–15 min, with extended breaks introduced as necessary to minimize hyperventilation symptoms in some patients. The interval IMT protocol is illustrated in Fig. 1 in the Supplement.

**Fig. 1:**
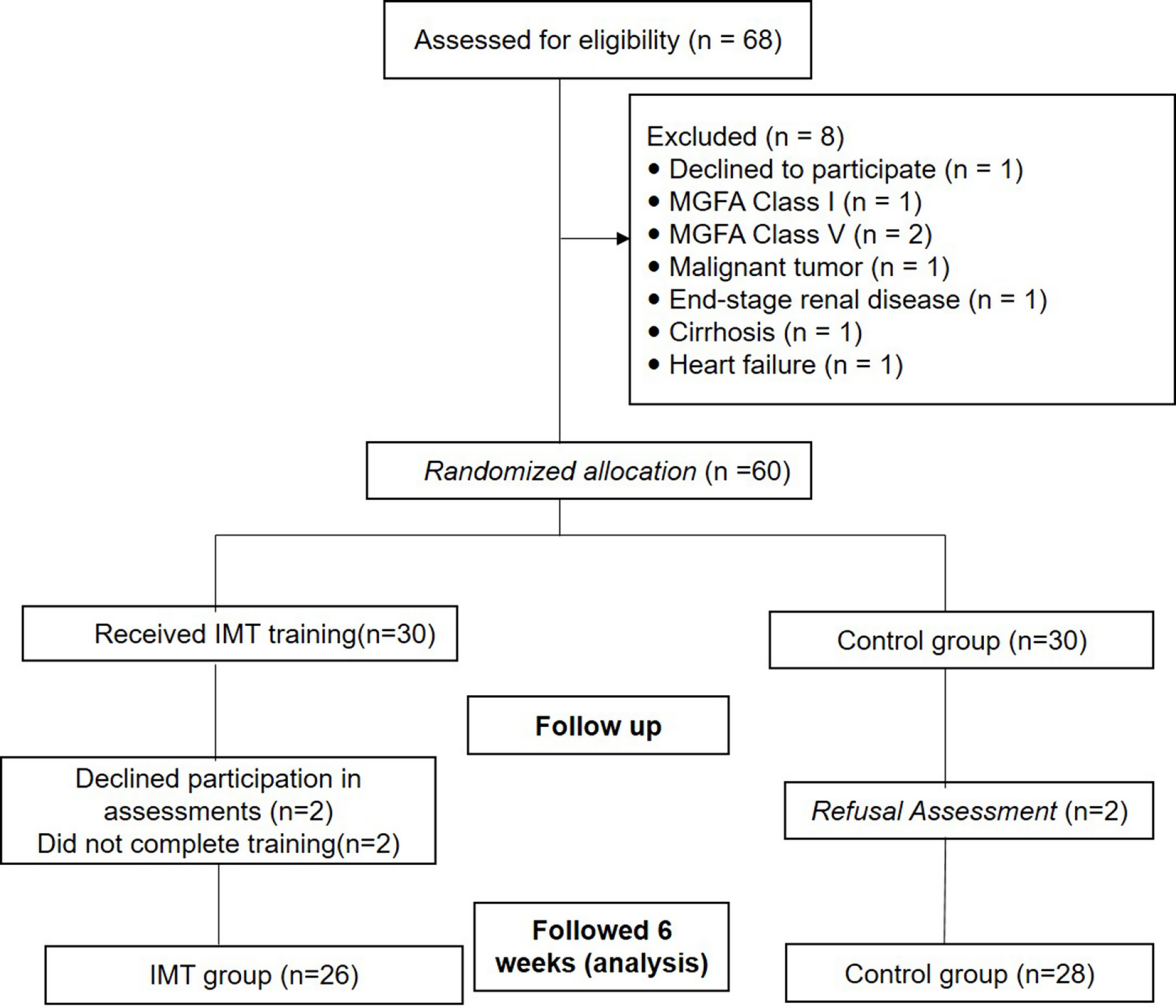
Study flowchart

The overall goal of the six-week IMT program was to achieve a moderate intensity level of aerobic exercise capacity. The initial loading pressure for the IMT device was set to 30% of the MIP of each participant. Following the principle of sequential progression of IMT, the load pressure was adjusted biweekly at the outpatient clinic. The load progressively increased, reaching 35% of MIP in the second week, 40% in the fourth week, and 45% in the sixth week.

In addition, aerobic exercise training consisted of upper limb exercises (raising arms without resistance), lower limb stepping warm-up, and walking exercises (5-minute slow walking, 2- minute brisk walking, followed by 5-minute slow walking) for 30 min per day. The aerobic exercise intensity was maintained at 50–70% of the participant’s maximum heart rate and monitored using a pulse oximeter. Walking speed was measured using a metronome as a guide to ensure consistent pace during home-based training. Furthermore, breathing control training was performed twice daily, including pursed-lip and diaphragmatic breathing, lasting for 10–15 min per session.

To ensure adherence to the exercise protocol, training compliance was monitored through self-reported training diaries, regular phone calls from a research nurse on alternate days, and biweekly reminders during outpatient clinic visits. Training diaries were structured as checklists to track the completion of warm-up exercises, walking sessions, breathing control exercises, and inspiratory muscle training. The participants were also educated on strategies for monitoring progress and managing any potential discomfort or adverse effects.

### Outcome measurements

The assessors were blinded to the randomization status of the patients during the outcome evaluation. Measurements were conducted at baseline and week 6 of the study. Demographic data, including age, sex, body height, body weight, body mass index, smoking status, MGFA classification, number of plasmapheresis sessions, and thymectomy history, were collected from both groups at baseline. Patients who underwent thymectomy were documented, irrespective of the timing of the procedure.

The primary outcome was respiratory muscle strength measured using MIP. Secondary outcomes included pulmonary function, dyspnea severity, expiratory muscle strength, and physical capacity on the six-minute walk test. Additionally, to account for the natural recovery of muscle strength following hospitalization, changes in these parameters from baseline to the end of the 6-week study period were calculated and analyzed for comparison. The MIP and maximum expiratory pressure (MEP) were assessed using a pressure manometer (Galemed Inc., Taiwan). The measurements were performed by two respiratory therapists who were randomly assigned to the patients. MIP and MEP were measured by instructing the patient to inhale and exhale with maximum effort against the device, maintaining the pressure for at least 1.5 s. Both measurement procedures were repeated three times with a one-minute rest between each repetition. To minimize fatigue-related effects on outcomes, the respiratory therapist continuously monitored patients through objective observations and verbal inquiries. Breaks were provided to maintain the measurement accuracy and reliability if fatigue was observed or reported.

The modified dyspnea Borg scale score was used to evaluate the level of breathing difficulty based on the patient’s self-perception of respiratory difficulty. The scores ranged from 0 (no respiratory difficulty) to 10 (extreme respiratory difficulty), with higher scores indicating more severe breathing difficulty. The modified Borg scale was recorded before measuring the MIP at both the baseline and the six-week mark.

The six-minute walk test was used to assess a patient’s exercise capacity and endurance according to the American Thoracic Society guidelines [14]. Before the test, patients were instructed to rest for at least 10 min before the test. During the test, patients were asked to walk as quickly as possible. Pulse oxygen saturation levels, heart rates, and modified Borg scale scores were monitored and recorded before, during, and after completion of the test.

Pulmonary function tests in this study strictly adhered to the guidelines of the American Thoracic Society using portable spirometry (Vyaire Spirometer, CareFusion Inc.). Pulmonary function tests for each patient included forced vital capacity (FVC), forced expiratory volume at one second (FEV_1_), FEV_1_/FVC ratio, and peak expiratory flow . The FVC was obtained by having the patient take a deep breath and then exhale forcefully. The test results are based on three reproducible measurements. Predicted values were calculated based on the patient’s age and height.

### Statistical analysis

The data were analyzed using SPSS 26.0, and the effect size was determined using the G-Power software. The chi-squared test was used to compare categorical variables between the two groups. Mann-Whitney U tests were used for nonparametric continuous variables. The Wilcoxon rank-sum test was used to assess improvements in each group from baseline to six weeks post-discharge. Statistical significance was set at *p* < 0.05, indicating a significant difference.

## Results

### Patient characteristics

A total of 68 patients discharged with MG were initially assessed. Figure 1 illustrates the flow of participant enrollment. Ultimately, 54 patients (26 in the IMT group and 28 in the control group) were included in the final analysis. The trial was concluded after reaching a predetermined sample size. The statistical power was recalculated using MIP along with the final results, and the sample size achieved a power level of 0.78. None of the participants reported experiencing any adverse effects during the intervention.

The baseline patient characteristics are shown in Table 1. At the time of enrollment, there were no significant differences in the physical condition and MG disease status between the two groups. On average, patients had been diagnosed with MG for 11 years, and there was a higher prevalence in women than in men with an elevated incidence of smoking history.

**Table 1:** Baseline characteristics of patients

	IMT group (*n* = 26)	Control group (*n* = 28)	*P*- value
Age (yr)	54.9 ± 15.2	56.21 ± 13.4	0.74^a^
Body high (cm)	161.1 ± 9.1	157.77 ± 11.56	0.24^a^
Body weight (kg)	64.9 ± 12.7	65.1 ± 16.6	0.97^a^
BMI (kg/m^2^)	25.0 ± 3.8	25.6 ± 3.8	0.54^a^
Disease duration (years)	11.5 ± 10.6	11.2 ± 8.7	0.93^a^
Sex			
Woman, n (%)	14 (53.8)	20 (71.41)	0.23^b^
Smoke			
Yes, n (%)	22 (84.6)	24 (85.7)	0.83^b^
MGFA classification			
IIA, n (%)	10 (38.5)	9 (32.1)	0.06^b^
IIB, n (%)	7 (26.9)	3 (10.8)	
IIIA, n (%)	5 (19.2)	8 (28.6)	
IIIB, n (%)	4 (15.3)	8 (28.6)	
Plasmapheresis			
Yes, n (%)	18 (69.2)	14 (50)	0.09^b^
Thymectomy			
Yes, n (%)	10 (38.5)	16 (57.1)	0.14^b^

Notes: Data are expressed as the mean ± standard deviation; ^**a**^ statistical analysis was performed using the Mann-Whitney test; ^**b**^ statistical analysis was performed using the *X*^2^ test

Abbreviations: IMT, inspiratory muscle training; BMI: Body mass index; MGFA, Myasthenia Gravis Foundation of America

The primary outcomes and other muscle strength measurements are presented in Table 2. The predicted values of MIP, MEP, and 6-minute walk distance were calculated according to the participants’ age and height based on a previous study [15]. Given which nature that all participants exhibited enhanced muscle strength and increased walking distance. At the 6-week mark, At the 6-week mark, both groups demonstrated significant improvements in MIP, modified Borg scale scores, and 6-minute walk distance. Notably, only the IMT + AE group showed significant improvement in MEP. The improvements in MIP were 33.8 ± 36.1 and 22.1 ± 25.8 cmH_2_O in the IMT + AE and control groups, respectively. The differences between the changes in MIP were not significant (*P* = 0.18). However, the improvements in the IMT + AE group were more significant in the MEP, modified Borg scale, and 6-minute walk distance.

**Table 2:** Comparisons of outcomes between two groups

Variable	Predicted	IMT group (*n* = 26)				Control group (*n* = 28)					Difference
		Baseline (%predicted)	6 weeks	Change; Δ_1_^a^	Change%; Δ_1_^a^	Predicted	Baseline (%predicted)	6 weeks	Change; Δ_2_	Change%; Δ_2_^a^	Δ_1_- Δ_2_^b^ Δ_1%_- Δ_2%_^b^
MIP (cmH_2_O)	73.2 ± 5.1	89.2 ± 49.7 119 ± 64%	123.0 ± 37.8	33.8 ± 36.1 *P* < 0.001^§^	57.0 ± 74.2%	73.0 ± 4.8	83.9 ± 43.6 117 ± 59%	108.5 ± 46.7	22.1 ± 25.8 *P* < 0.001^§^	46.3 ± 71.3	*P* = 0.18 *P* = 0.57
MEP (cmH_2_O)	98.3 ± 6.6	77.7 ± 22.3 97 ± 35%	98.5 ± 29.9	20.8 ± 20.1 *P* < 0.001^§^	15.8 ± 29.3	98.1 ± 6.2	91.4 ± 38.3 94 ± 6%	92.8 ± 37.9	1.4 ± 15.3 *P* = 0.62	4.9 ± 25.9	*P* < 0.001^§^ *P* = 0.01^*^
modified Borg scale		1.27 ± 0.82	0.57 ± 0.59	1.50 ± 0.88 *P* < 0.001^§^			2.11 ± 0.86	1.29 ± 0.88	0.82 ± 0.83 *P* < = 0.03^*^		*P* = 0.03^*^
6MWD (meter)	498.2 ± 105.3	374.0 ± 59.4 78 ± 18%	481.9 ± 64.3	107.9 ± 72.1 *P* < 0.001^§^	21.7 ± 26.5	481.3 ± 102.9	386.8 ± 96.9 81 ± 22%	412.4 ± 86.4	25.6 ± 46.8 *P* = 0.03^*^	13.3 ± 25.9	*P* = 0.003^*^ *P* = 0.03^*^

^a^ Δ_1_, Δ_2_: Changes from the baseline to the 6th week, compared with paired t-test; ^b^ Δ_1−_Δ_2_: difference in changes between two groups, compared with Mann-Whitney test; change%: difference in percentage changes, compared with Mann-Whitney test; ^*^P < 0.05; ^§^*P* < 0.001

Abbreviations: IMT, inspiratory muscle training; MIP, maximum inspiratory pressure; MEP, maximum expiratory pressure; 6 MWD, 6-minute walk distance

Table 3 presents the results of the pulmonary function tests. The IMT + AE group reported improvements in FVC and FVC % of prediction (0.21 ± 0.24 L and 6.17 ± 6.01%, respectively), while the control group revealed a declined FVC (-0.06 ± 0.30 L and − 1.79 ± 9.69%, respectively) after 6 weeks of discharge. The difference in the changes between the two groups was significant (*P* < 0.001). The improvement of the FEV_1_ value was significant with IMT + AE (0.19 ± 0.34) vs. reduction in the control group (-0.05 ± 0.28 L); however, the difference in changes in % of prediction did not reach significance (*P* = 0.21). Changes in forced expiratory flow in FEV_1_/FVC (%) and peak expiratory flow were insignificant within and between the groups (*P* = 0.72).

**Table 3:** Comparisons of pulmonary function test

Variable	IMT group (*n* = 26)			Control group (*n* = 28)			Difference
	Baseline	6 weeks	Change; Δ_1_^a^	Baseline	6 weeks	Change; Δ_2_^a^	Δ_1_- Δ_2_^b^
FEV_1_ (L)	2.01 ± 0.74	2.21 ± 0.70	0.19 ± 0.34 *P* = 0.01^*^	1.79 ± 0.71	1.74 ± 0.71	-0.05 ± 0.28 *P* = 0.41	*P* < 0.001^§^
FEV_1_ (%_pred_)	75.01 ± 18.21	78.5 ± 21.2	6.17 ± 1.79 *P* = 0.01^*^	76.08 ± 14.4	73.12 ± 19.1	-1.79 ± 9.69 *P* = 0.01	*P* = 0.21.
FVC (L)	2.57 ± 0.85	2.77 ± 0.84	0.21 ± 0.24 *P* = 0.01^*^	2.45 ± 0.88	2.39 ± 0.94	-0.06 ± 0.30 *P* = 0.30	*P* < 0.001^§^
FVC (%_pred_)	75.03 ± 18.4	81.2 ± 17.91	6.17 ± 6.01 *P* < 0.001^§^	76.0 ± 17.9	74.283 ± 18.14	-1.79 ± 9.69 *P* = 0.01	*P* = 0.001^*^
FEV_1_/FVC (%)	77.92 ± 9.74	79.22 ± 5.69	1.30 ± 10.38 *P* = 0.08^*^	72.41 ± 10.72	74.27 ± 9.93	1.86 ± 4.66 *P* = 0.07	*P* = 0.72
PEF (L/s)	5.04 ± 1.71	5.28 ± 2.21	0.24 ± 1.47 *P* = 0.41	4.54 ± 1.96	4.06 ± 1.93	-0.48 ± 1.06 *P* = 0.02	*P* = 0.09

^a^ Δ_1 or_Δ_2_: Changes from the baseline to the 6th week, compared with paired t-test; ^b^ Δ_1−_Δ_2_: the difference of two of changes between two groups, compared with Mann-Whitney test; ^*^P < 0.05; ^§^*P* < 0.001

Abbreviations: IMT, inspiratory muscle training; MEP, maximum inspiratory pressure; MEP, maximum expiratory pressure; 6 MWD, 6-minute walk distance

Table 4 shows the changes in muscle strength and pulmonary function test results from baseline to 6 weeks between the groups, including the effect size and confidence interval for the comparisons. The IMT + AE group demonstrated significantly greater improvements in MEP (*P* < 0.001), modified Borg scale score (*P* < 0.001), 6-minute walk distance (*P* < 0.001), and FEV_1_ as a predicted percentage (*P* < 0.001). Additionally, the minimum clinically important difference (MCID) for statistically significant results was calculated using the following equation:

**Table 4:** Comparative changes in muscle strength and pulmonary function test results after 6 weeks MCID

Outcomes	IMT group (*n* = 26)	Control group (*n* = 28)	MD (95% CI)	*P* Value	Effect	MCID
MIP (cmH_2_O)	33.8 ± 36.1	33.8 ± 36.1	9.96 (-0.06–39.91)	0.05	0.54	26.8
MEP (cmH_2_O)	20.8 ± 20.1	20.8 ± 20.1	4.37 (19.21–28.04)	< 0.001^§^	1.14	25.4
Modified Borg Scale	1.50 ± 0.88	0.82 ± 0.83	-1.05 (-1.51 - -0.59)	< 0.001^§^	1.46	1.2
6MWD (meter)	0.82 ± 0.83	0.82 ± 0.83	64.12 (25.27–103.02)	0.002^*^	0.89	52.8
FEV_1 Pred_	0.82 ± 0.83	-0.05 ± 0.28	7.97 (3.37–12.56)	0.001^*^	0.95	0.7
FVC _Pred_	-0.48 ± 1.06	-0.06 ± 0.30	0.94 (-5.1–6.94)	0.75	0.18	
FEV_1_/FVC (%)	-0.05 ± 0.28	1.86 ± 4.66	0.55 (-4.89–3.79)	0.79	0.06	
PEF (L)	1.86 ± 4.66	-0.48 ± 1.06	0.61 (-0.11–1.32)	0.09	0.39	

Abbreviations: IMT, inspiratory muscle training; MIP, maximum inspiratory pressure; MEP, maximum expiratory pressure; 6 MWD, 6-minute walk distance; FEV_1_, forced expiratory volume in one second; FVC, forced vital capacity; PEF, peak expiratory flow; MD, mean difference; CI, confidence interval; MDIC, minimal clinically important difference. Comparisons were conducted using the t-test; ^*^P < 0.05; ^§^*P* < 0.001

MCID = Cohan’s *d* * standard deviation at baseline.

## Discussion

After six weeks of inspiratory muscle and exercise training for patients with MG, significant improvements were observed in expiratory muscle strength, dyspnea severity, exercise capacity, and pulmonary function. This study shows that a six-week pulmonary rehabilitation program employing IMT + AE effectively enhances physical function in individuals with MG.

### Improving respiratory muscle strength

Previous studies have shown that IMT strengthens respiratory muscles, counteracts muscle degeneration, and reduces respiratory distress in MG patients [4]. After six weeks, the IMT group demonstrated a substantial increase in MIP, improving by 33.8 cmH_2_O compared with 22.1 cmH_2_O in the control group. The positive outcomes align with findings from previous studies that endorse the effectiveness of interval-based IMT [13, 16]. Fregonezi et al. first demonstrated that IMT with 60% workload for 8 weeks improves the MIP and respiratory rate to tidal volume ratio among patients with MG [13]. While most studies reported statistical significance. Del Corral et al. reported minimum clinical differences of 18 cmH_2_O in individuals undergoing IMT with long-term post-COVID. The minimal clinically significant difference in IMT in patients with neuromuscular disease is warranted for further study [17]. Our results showed a minimum clinical difference of 26 cmH_2_O of MIP in patients with MG.

In contrast, expiratory muscle strength (MEP) exhibited more pronounced improvements in the IMT group, increasing by 20.8 cmH_2_O compared to 1.4 cmH_2_O in the control group. This finding suggests that IMT + AE effectively engages the expiratory muscles and enhances respiratory efficiency. The active involvement of expiratory muscles during training, particularly the abdominal and internal intercostal muscles, may contribute to their superior adaptability compared with inspiratory muscles. The physiological demands of exercise, especially the hyperventilation response triggered by increased CO₂ levels, further stimulate expiratory muscle activation, facilitate rapid air expulsion, and improve overall respiratory function [18, 19].

When a patient performs IMT training, the process of repeated respiratory loading not only strengthens the inspiratory muscles to increase the strength of the intercostal muscles and diaphragm muscles, but also encourages the expiratory muscles (e.g., rectus abdominis and internal intercostal muscles) and their endurance to participate in active expiration, which further improves the overall respiratory function. This phenomenon can be observed in athletes with enhanced MEP (31%) [20], improved maximal oxygen uptake, and improved anaerobic capacity after 4–12 weeks of IMT [18, 21]. Thus, IMT improves the strength of the intercostal and/or abdominal muscles, generating sustained contraction during exercise, which results in adequate ventilation and improved respiratory muscle efficiency. Reducing the oxygen supply to the intercostal and/or abdominal muscles during skeletal muscle-related exercise can provide greater resistance to fatigue throughout exercise.

Previous studies have reported significant associations between expiratory muscle pressure and functional capacity, and between expiratory muscle pressure and disease severity, indicating that patients with impaired respiratory muscle strength have lower functional capacity and more severe disease [10].

The expiratory muscle strength improvements in our study compared to the control groups are potentially attributable to the incorporation of aerobic exercises, pursed-lip breathing, and diaphragmatic breathing within our intervention. A previous study combining lower-limb endurance exercise with breath control demonstrated significant improvements in expiratory muscle pressure [22]. Therefore, improvements in expiratory muscle training might benefit from breathing maneuvers and aerobic exercises using core muscles.

### Enhanced in pulmonary function and fatigue reduction

Pulmonary function assessments revealed significant improvements in FEV_1_ and FVC following the IMT + AE intervention. Fernández-Lázaro et al. revealed that a resistance load of ≥ 15% of the MEP applied during IMT improved significantly (54%) within 4 weeks [13]. From week 6, maximal oxygen uptake improved significantly, reaching ≥ 21.5% of the MEP after IMT, proving IMT beneficial for pulmonary function. Given that patients with MG often experience impaired neurotransmission, leading to diminished respiratory muscle strength, these enhancements highlight the potential of IMT in mitigating disease-related pulmonary deficits.

Fatigue is a common symptom in patients with MG that often exacerbates respiratory inefficiency and increases the risk of myasthenic crises. The implementation of IMT improved respiratory muscle endurance, enabling patients to sustain adequate pulmonary ventilation despite a reduced neurological drive. Notably, the modified Borg scale scores showed significant improvement after six weeks of IMT + AE, with a more pronounced reduction in dyspnea severity observed in the intervention group. Hsu et al. reported that a 12-week IMT program significantly delayed muscle fatigue progression and reduced respiratory failure risk in patients with MG [23]. These is consistent with previous studies demonstrating that respiratory muscle training can delay muscle fatigue progression and reduce the risk of respiratory failure.

IMT has been associated with diaphragmatic hypertrophy, increased blood flow to respiratory muscles, decreased dyspnea perception, and optimized neuromotor control, all of which contribute to enhanced respiratory efficiency and endurance. Salazar-Martínez et al. reported IMT’s physiological effects of IMT, including diaphragmatic hypertrophy, increased blood flow to exercising muscles, decreased fatigue and dyspnea, increased respiratory efficiency and endurance, changes in muscle fiber composition, and optimized neuromotor control of respiratory muscles [24]. The observed benefits may be attributed to physiological adaptations such as an increased proportion of type IIa muscle fibers, which enhance resistance to fatigue and FRC.

Previous investigations have consistently demonstrated compromised pulmonary function in individuals with MG, particularly in reduced FEV_1_ levels [19]. Similarly, Zawadka-Kunikowska et al. reported significantly lower FEV_1_ and FVC values in MG patients compared to healthy controls [25]. A related study exploring the efficacy of IMT over 12 weeks noted that improvements in both FEV_1_ and FVC alongside reductions in fatigue were appeared among MG patients [26].

This enhancement may be attributed to increased expiratory muscle strength. The IMT program aims to enhance expiratory muscle strength, leading to an increased FEV_1_. A previous study demonstrated a correlation between MEP, and six-minute walk distance, and peak expiratory flow in patients with multiple sclerosis [25].

### Impact on exercise capacity and dyspnea scores

Disease severity in MG can affect respiratory status, as reflected in patients’ dyspnea scores. Generally, patients with mild severity experience dyspnea during exercise or exertional activity, whereas those with a worsening condition have an intense sensation of dyspnea. In severe cases, dyspnea may occur during sleep [25]. The six-minute walk test and dyspnea severity assessments provided further evidence of the efficacy of the intervention. Patients in the IMT + AE group exhibited significant improvements in walking distance, reflecting an enhanced exercise capacity. Dyspnea scores, which serve as important indicators of disease severity, were markedly lower in the intervention group than in the control group. These findings suggest that IMT + AE contributes to meaningful improvements in the functional capacity and respiratory endurance of patients with MG.

Existing studies emphasizing long-term inspiratory muscle strength training have shown positive outcomes, often requiring extended durations to yield noticeable benefits [22, 26]. However, our intervention with a condensed six-week duration exhibit pronounced improvements in the 6-minute walk test and exercise capacity when compared to similar studies [8, 27]. This condensed training regimen may represent a paradigm shift in respiratory rehabilitation for patients with MG, offering a time-efficient alternative to traditional long-term protocols.

### Benefits of utilizing intermittent training among MG patients

The intermittent nature of the IMT + AE training program proved advantageous in enhancing respiratory endurance and pulmonary function, while improving adherence to exercise regimens. Incorporating rest intervals allowed patients with MG to sustain higher training intensities without experiencing excessive fatigue, thereby maximizing the benefits of respiratory muscle conditioning.

Clinical observations indicate that many patients with MG exhibit psychological or perceptual reluctance toward physical activity, often resulting in reduced daily activity levels and diminished tolerance for sustained exertion. Continuous exercise protocols may exacerbate muscle fatigue, potentially compromising respiratory performance, and increasing the risk of respiratory failure. Physiologically, prolonged training sessions impose significant metabolic demands on respiratory muscles, leading to cumulative fatigue and impaired endurance. This phenomenon may be linked to the respiratory muscle metabolic reflex, wherein fatigued respiratory muscles signal the central nervous system via type III and type IV sensory nerves, triggering a sympathetic vasoconstrictor response that exacerbates peripheral muscle fatigue and increases perceived exertion [28, 29].

Intermittent IMT addresses these challenges by alternating moderate-intensity exercise with rest periods, effectively mitigating excessive fatigue and optimizing pulmonary ventilation. This structured approach enhanced exercise tolerance, promoted adherence, and reduced the likelihood of training-induced respiratory distress. Given these advantages, intermittent IMT may serve as an effective strategy to improve respiratory function and overall well-being in patients with MG.

### Limitations

This study had specific limitations that must be considered when interpreting the results. This single-center nature may affect the generalizability of our findings. Participants were recruited at their initial outpatient clinic visits after discharge. It is possible that the medication regimens were altered to enhance muscle strength. The patient’s condition was managed under the same treatment protocol; however, the extent to which these medications influence the outcomes remains unclear. Furthermore, training compliance was monitored using self-reported diaries, phone calls on alternate days, and biweekly reminders during outpatient clinic visits; however, these methods are still subject to bias. MG’s progressive nature of MG may also lead to muscle strength fluctuations over time, potentially impacting the long-term sustainability of IMT benefits. Future studies should incorporate objective assessment tools such as fatigue severity scales to enhance the accuracy of evaluating respiratory muscle fatigue and training adherence. In addition, wearable devices can be used to monitor adherence to the training protocol more effectively.

### Clinical implications and future suggestions

Based on our findings, we recommend the integration of an individualized IMT training strategy into respiratory rehabilitation programs for MG patients. A structured approach beginning with low-intensity IMT (30% of MIP) and gradually increasing resistance based on patient fatigue levels may optimize therapeutic outcomes while minimizing the risk of overexertion. Implementing an intermittent IMT strategy consisting of six sets of five breaths (30 breaths total) performed twice daily may further enhance training efficacy by preventing hyperventilation and excessive fatigue.

The use of real-time monitoring tools may provide valuable insights into respiratory fatigue patterns and facilitate personalized training adjustments. Future research should explore the applicability of intermittent IMT + AE in patients with more severe MG, particularly during the recovery phase following myasthenic crisis. Longitudinal studies examining the long-term effects of IMT on hospitalization rates, disease progression, and survival outcomes are warranted to elucidate the clinical utility of respiratory muscle training in MG management.

## Conclusion

In conclusion, this study demonstrated that a six-week IMT + AE program significantly improved respiratory muscle pressure, dyspnea severity, exercise capacity, and pulmonary function in patients with MG. The combination of moderate-intensity interval IMT + AE would be an effective strategy for post-hospitalization recovery, potentially enhancing long-term respiratory function and quality of life. Implementing IMT as an early intervention following hospital discharge may benefit patients with MG, reinforcing the need for continued research on objective monitoring and long-term efficacy.

## Electronic supplementary material

Below is the link to the electronic supplementary material.

Supplementary Material 1: Supplement Figure 1 Interval Inspiratory Muscle Training Protocol. The interval training protocol for inspiratory muscle training consists of a series of breath cycles that incorporate variations in breath-holds and breaks between breaths

## Data Availability

The data supporting this research are available from Hui-Ling Lin.

## Abbreviations

MG: Myasthenia gravis
IMT + AE: Inspiratory muscle training and aerobic exercise
FVC: Forced vital capacity
FEV_1_: Forced expiratory volume at one second
MIP: Maximal inspiratory pressure
MEP: Maximal expiatory pressure
