# Supplementary figures and images for: Inspiratory muscle training and aerobic exercise for respiratory muscle strength in myasthenia gravis post-hospitalization- a randomized controlled trial
Source: BMC Pulm Med. 2025 May 27;25:266. doi: 10.1186/s12890-025-03733-7 (PMC12107784; doi:10.1186/s12890-025-03733-7)

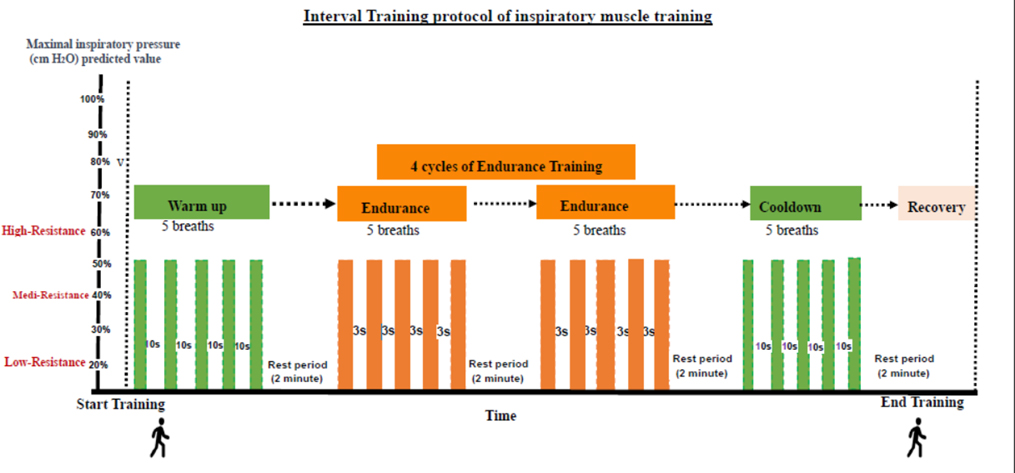

Supplement: Supplementary file 1 — Supplementary Material 1: Supplement Figure 1 Interval Inspiratory Muscle Training Protocol. The interval training protocol for inspiratory muscle training consists of a series of breath cycles that incorporate variations in breath-holds and breaks between breaths [file 12890_2025_3733_MOESM1_ESM.jpg]
